# Cognitive Reserve Moderates the Impact of Depression on Cognitive Performance in Aging

**DOI:** 10.1093/geroni/igaf122.3898

**Published:** 2025-12-31

**Authors:** Rabia Khalaila

**Affiliations:** Zefat Academic College, Zefat, HaZafon, Israel

## Abstract

depression and cognitive performance in older adults and explores how cognitive reserve (CR)—measured through a composite score of education, occupational complexity, and cognitive activities, as well as each individual component—moderates this relationship over a four-year period.

**Methods:**

Data from 32,325 participants aged 50+ in the Survey of Health, Aging, and Retirement in Europe (SHARE) were analyzed. Cognitive performance was measured using memory, numeracy, and verbal fluency tests at baseline and after four years. Depressive symptoms were assessed with the EURO-D scale, and CR was evaluated through education, occupational complexity, and cognitive activity engagement. Moderation effects were tested using bootstrapping with resampling strategies.

**Results:**

Lower and average CR levels were linked to a stronger negative association between depression and cognitive performance, while higher CR showed no adverse effects. Education and cognitive activities significantly reduced depression’s impact on cognitive function, whereas occupational complexity had no significant effect.

**Conclusions:**

Cognitive reserve, particularly through education and cognitive activities, moderates the impact of depression on cognitive performance in older adults. Public health strategies should promote CR-enhancing activities to protect cognitive health in later life.

